# Linguistic Features of Older Adults’ Daily Self-Talk: A Naturalistic Observational Approach

**DOI:** 10.1093/geroni/igaf122.3804

**Published:** 2025-12-31

**Authors:** Sibo Gao, Karen Fingerman

**Affiliations:** University of Texas at Austin, Austin, Texas, United States; The University of Texas at Austin, Austin, Texas, United States

## Abstract

Research supporting socioemotional selectivity theory finds that older adults tend to have better emotion regulation than younger adults, particularly with regard to negative emotion. Verbalized self-talk (i.e., audible speech when alone) may be an important but understudied emotion regulation strategy in late life, promoting psychological distance through second-person pronouns and structured speech, and providing an opportunity to express negative emotions. The current study sought to characterize linguistic features of verbalized self-talk descriptively and in comparison to conversations with social partners. Older adults (N = 333; Mage=73.9) reported whether they had been with social partners every three hours across 5–6 days. Electronically Activated Recorders captured 30-second sound snippets every 7 minutes. Coders transcribed 109,609 snippets; 918 snippets contained verbal utterances when participants were alone (self-talk). Using the Linguistic Inquiry and Word Count, we extracted linguistic features including second-person pronouns, formal and structured language, and emotional tone. Multilevel models revealed no significant within-person association between being alone and second-person pronoun use. However, when participants were alone, they used more structured language and expressed more negative emotion compared with language when with social partners. This study makes an important contribution by capturing naturalistic self-talk as it occurs in daily life and suggests self-talk may help older adults regulate negative emotions. Future research using wearable sensors might capture arousal or allow self-reported experience sampling of unspoken self-talk to tease out when and who uses self-talk to regulate emotions.

